# Coping strategies to manage acculturative stress: Meaningful activity participation, social support, and positive emotion among Korean immigrant adolescents in the USA

**DOI:** 10.3402/qhw.v7i0.18870

**Published:** 2012-11-19

**Authors:** Junhyoung Kim, Wonseok Suh, Sooyeon Kim, Himanshu Gopalan

**Affiliations:** 1 Department of Human Performance and Sport Sciences, Winston-Salem State University, Winston-Salem, NC, USA; 2 Department of Creative Informatics & Computing Institute, Korea University, Seoul, South Korea; 3 School of Tourism & Food Service, Yeungnam College of Science and Technology, Dae-Gu, South Korea

**Keywords:** Acculturative stress, adaptation, coping strategy, Korean immigrant adolescents, meaningful activity

## Abstract

During acculturation, Asian immigrant adolescents have numerous challenges such as language barriers, cultural and ethnic differences, different school environments, discrimination experiences, and intergroup conflicts and tension. These challenges generate acculturative stress, which negatively affects the perception of health and well-being among Asian immigrant adolescents. This article explored how Asian immigrant adolescents perceive and cope with acculturative stress. In particular, this study examined the stress-coping strategies in the adaptation process as experienced by Korean immigrant adolescents. Three main themes associated with the stress-coping strategies were captured: (a) engagement in meaningful activities; (b) social support; and (c) positive emotion. This finding implies that Asian immigrant adolescents create and develop their own strategies to deal with acculturative stress, which results in a sense of happiness and psychological well-being. This study discuss the future implications on how to improve the perception of health and well-being among Asian immigrant adolescents.

It is well known throughout literature that Asian immigrant adolescents perceive and deal with serious acculturative stress because of a multitude of challenges, such as communication problems, discrimination experiences, different school environments, interracial conflicts and tension, and academic works (Ayers et al., 2009; Fischer, 2010; Yeh, 2003; Yu, Huang, Schwalberg, Overpeck, & Kogan, 2003). Such stress related to acculturation causes negative psychological symptoms such as depression, anxiety, and social isolation for Asian immigrants (Lin & Yi, 1997; Yeh & Inose, 2002).

Among Asian immigrant adolescents, Korean immigrant adolescents, in particular, have numerous challenges associated with the acculturation process. The critical issue that Korean immigrant adolescents face is conflicts with their parents because of different degrees of acculturation (Kim, 1997; Moon, 2008). In a Korean family structure, parents emphasized the value of Confucianism focused on filial piety, family ties, and the patriarchal family order (Min, 1998) and parents in Western cultures focused on independence and individuality in their children (Jo, 1999). During acculturation, Korean immigrant adolescents are easily adapted with new cultural values and beliefs (e.g. independence and individualism), but their parents tend to maintain their Asian cultural perspectives (e.g. Confucianism) (Kim, 1997). Yeh et al. (2005) examined the cultural negotiation process faced by Korean immigrant adolescents and discovered that when they struggle to balance within themselves the “American” and “Korean” cultures, their peer and parent relationships are negatively affected.

In addition, different levels of acculturation between Korean immigrant adolescents and their parents generate divergent views on culturally relevant issues, such as academic achievement, culturally appropriate behavior, respect for parents and the elderly, and familial obligations (Jo, 1999; Min, 1998; Moon, Wolfer, & Robinson, 2001). For example, Moon (2008) argued that Korean American parents have “high expectations of academic and occupational achievement which can create a great tension in Korean American homes” (p. 229). In addition, Asian American parents have a strong tendency to control their children in order to secure their academic success (Rohner & Pettengill, 1985; Storm, Park, & Daniels, 1987) and demand higher academic grades from their children than white parents (Jo, 1999; Yao, 1985). Such parents’ expectations of academic performance may provide additional stress and psychological burdens to Korean immigrant adolescents. If they fail to satisfy their parents’ expectations, they may be likely to experience psychological distress and stress. Thus, Korean immigrant adolescents may deal with numerous challenges and culture-related issues that other ethnic groups might not experience.

In a school setting, Korean immigrant adolescents experienced a variety of stressors such as racial discrimination, racial or ethnic stereotyping, language barriers, and intergroup conflicts and tensions (Chiu & Ring, 1998; Yeh, Ching, Okubo, & Luthar, 2007; Yeh & Inose, 2002). Although they have been reported to experience higher academic success and fewer delinquent behaviors than other racial groups (Lorenzo, Frost, & Reiunharz, 2000), Asian immigrant adolescents have been found to have higher levels of negative psychological symptoms, such as depression, isolation, and anxiety (Lorenzo et al., 2000; Wong, 2001). Moreover, a recent study of Korean immigrant adolescents found that acculturative stress and difficult adaptation processes have been associated with lower levels of happiness and higher levels of negative psychological symptoms and suicidal ideation (Cho & Haslam, 2010; Shin, Han, & Kim, 2007). Thus, stresses associated with the adaptation process affect immigrant adolescents’ lives in diverse and negative ways.

In terms of mental health among Korean immigrant adolescents, previous research described that they have particular and unique mental health concerns such as high expectation of living in a new country, culture shock, acculturative stress, and adaptation difficulties (Cho & Haslam, 2010; Homma-True, 1997; Kim, 1997; Yeh, 2003). According to Kim (1996), Korean immigrant adolescents have high expectations of what their new lives will be once they move to the United States. When they face numerous challenges and obstacles after immigration and their expectations are not fully satisfied, Korean immigrant adolescents experience culture shock, depression, anger, resentment, and disappointment (Kim, 1996). In a recent study by Cho and Haslam (2010), four groups were created to examine suicidal ideation and distress: (1) Korean students living with both parents in the United States (Korean immigrants) intending to live there permanently; (2) Korean students living alone or with one parent in the United States (Korean internationals) for the primary reason of studying; (3) Korean students living in Korea (Korean indigenous); and finally (4) non-Korean American students living in the United States (American indigenous). Their findings demonstrate that Korean immigrants and Korean international students have higher levels of life stress, distress, psychological symptoms, and suicidal ideation than other groups.

To deal with various stressors, much research has emphasized the role of coping strategies as any individuals’ resources (Lazarus & Folkman, 1984; Zeidner & Endler, 1996). Lazarus and Folkman (1984) defined stress-coping as “constantly changing cognitive and behavior efforts to manage specific external and/or internal demands that are appraised as taxing or exceeding the resources of the person” (p. 141). The notion that the cognitive and behavioral efforts are constantly changing to manage the situation implies that individuals attempt to utilize all resources related to coping resources in order to determine which strategies will work best in different situations. Folkman and Moskowitz (2004) mentioned that there are some challenges to understand in regard to stress-coping process and outcomes. They provided the idea that there are other variables that affect stress-coping such as social aspects of coping, religious coping, emotional regulations, positive thoughts and minds, and future-oriented dimensions. The past research lacks evidence to show how these variables are related to stress-coping.

Therefore, it is important for health care professionals to understand how Korean immigrant adolescents manage and cope with a variety of stress associated with acculturation. If health care professionals are more aware of the coping strategies that Korean immigrant adolescents develop, they are more likely to provide effective coping resources for Asian immigrant adolescents who are experiencing distressing problems and negative psychological symptoms because of adaptation difficulties. However, little information exists about how Korean immigrant adolescents perceive and cope with acculturative stress. Thus, this study utilized a qualitative research approach to explore ways in which Korean immigrant adolescents cope with acculturative stress.

## Methods

The research design has a qualitative approach that employed in-depth interviews. Our approach was a dynamic and iterative process that generated in-depth understanding, and one which obtained individuals’ interpretations of their experiences and probed their life experiences (Crabtree & Miller, 1999). In-depth interviews are useful to specify variations of a phenomenon and its relevant conditions (McMillan & Schumacher, 2001). According to Hesse-Biber and Leavy (2006), in-depth interviews are appropriate and useful when the researcher has a particular topic that he or she wants in order to obtain deep information and probe life events. As described earlier, in-depth interviews are helpful when this study explores Korean immigrant adolescents and how they deal with their immigration lives. In addition, these in-depth interviews are useful for accessing subjugated voices and discovering subjugated experiences, such as those of women, people of color, homosexuals, and the poor (Hesse-Biber & Leavy, 2006). Korean immigrant adolescents belong to the ethnic groups that are excluded from an understanding of their own life stories. Therefore, in-depth interviews should enable us to access their hidden knowledge and probe their particular experiences.

The aim of this study was to capture the coping strategies of Korean immigrant adolescents and how they deal with acculturative stress. It focused on describing and interpreting the experiences and meanings associated with stress-coping due to acculturative stress.

### Participants

This article used purposeful criteria-based sampling strategies (Strauss & Corbin, 1998). The criteria for the participants required the individuals to: (a) have immigrated to the United States from South Korea; (b) be in attendance of a middle or high school in the United States; and (c) identify their ethnic identities as Koreans. Recruitment occurred in cooperation with the members of Korean communities (e.g. church and local Korean organizations) located in northeastern United States. During the meeting with the members of Korean communities, we introduced the information related to this study, that is, the objective and criteria for participants for the study, and distributed the flyers to recruit potential participants. After 2 weeks, the potential participants contacted us to gain more information on this study via email and/or telephone. Among those who expressed an interest in participating, the research team contacted them and explained the study (e.g. purpose, confidentiality, time-frame, waiver information, and contact information). The university ethnic board approved these procedures.

The 18 potential participants were informed about the ethnic considerations of confidentiality that participation was voluntary and at any time they were able to withdraw from the study. The 15 final participants voluntarily agreed to participate in this study with informed consents. Of these participants, nine were men and six women. Four were middle school students and 11 were attending high school. They ranged in age from 13 to 18. Thirteen of the participants were staying with a host family because their parents were still living in Korea. These 13 were born in the United States while their parents were studying at universities and decided to come back to United States for their own studies. The other two participants lived with their parents. The average length of time since the participant's had immigrated was 32 months.

### Data collection and procedure

This study used the semistructured interviews to obtain information about the participants’ knowledge, feelings, and experiences associated with coping resource and strategies. The interviewer (first author) interviewed each participant at his or her home and audiotaped the interview. Each interview lasted 45–90 min. At the beginning of each interview, the interviewer introduced himself and briefly described the purpose of the study. The researcher team developed a total of 10 open-ended questions. They also designed a series of probes, which helped participants elaborate on their experiences associated with stress-coping strategies. The interviewer asked grand-tour questions to allow them to share their life challenges associated with acculturation. Subsequently, the interviewer asked mini-tour questions to explore ways in which participants coped with stress and how they determined their coping strategies. The questions asked were: “How could you react to the various stressors that you described?”, “What is your way of relieving stress when you feel stressed?”, “What resources do you utilize to manage your stress?”, and “Please let me know if you experience successful strategies for coping with stress”. The final questions were related to a sense of happiness among participants: “Tell me about your overall happiness now” and “What contributes to your happiness?”.

At the end of the interview, the participants completed a demographic survey that asked their gender, their age, and their length of stay. Immediately following each interview, the interviewer recorded field notes, including personal insights and participants’ observed emotional changes, behaviors, and communication skills as well as the environmental conditions of the interview (Strauss & Corbin, 1998). To clarify the transcript and interpretations of their experiences, the research team used follow-up phone calls and emails to communicate with the participants.

### Data analysis

A thematic analysis was used so as to explore how participants deal with acculturative stress to enable us to “… increase their accuracy or sensitivity in understanding and interpreting observations and interviews about people, events and situations” (Boyatzis, 1998, p. 5). This study was followed with the data collection and analysis of qualitative research introduced by Creswell (2009). The first step was for the interviewer (first author) to create each interview transcript taking into consideration the field notes. With the first draft of the transcript, the researchers (the second and third authors) reviewed this whilst listening to each recording to verify the accuracy of the transcript. The next step was for the research team to organize and prepare the data for analysis. In order to accurately understand and interpret the participants’ experiences, the research team thoroughly read the transcripts and subsequently created an open-coding scheme for each transcription. The research team then compared and contrasted the patterns of participants’ experiences associated with coping strategies. Using the themes and quotes that emerged from the data, the research team interpreted and verified the themes associated with our study purposes and analyzed the meanings and identified the core themes. When discrepancies among researchers emerged, the research team discussed them until they were resolved and where necessary, they shared identified themes with participants in order to clarify the discrepancies. After analyzing the core themes, the research team contacted the participants to verify the accuracy of the data and gained agreement from them.

### Trustworthiness in qualitative research

To improve the validity and reliability of the qualitative data, qualitative researchers introduced various techniques such as the expert review, the member checking process, and the negative case analysis (Creswell, 2009; Kvale, 1996; Lincoln & Guba, 1999). In this study, expert review process and member checking process were used to increase trustworthiness and rigor in data analysis. In the process of member checking, the research team contacted participants to verify the interpretations and data gathered during their interviews (Erlandson et al., 1993). The research team provided them with a summary of the themes (Peterson et al., 2007). In terms of expert review, the research team was involved in the coding process in order to reach an agreement associated with the findings of the themes and interpretation of the data (Lincoln & Guba, 1999).

## Results

This study was identified with three key themes from the qualitative data that captured the coping resources and coping strategies utilized by Korean immigrant adolescents: (a) engagement in meaningful activities; (b) social support; and (c) positive emotions. These main themes were identified based on the participants’ experience since immigration.

### Engagement in meaningful activities

Participation in meaningful activities was the most salient theme to emerge from the data associated with the coping strategy. Most participants were engaged in a variety of activities such as club activities, volunteer work, and hobbies as a way of coping with acculturative stress. By engaging in meaningful activities, they felt that they enhanced their sense of happiness and enjoyment in their lives, expanded social connections with other ethnic groups, and reduced some levels of stress related to living apart from their family.

Some of the participants believed that participation in club activities was an effective coping strategy to cope with stress related to limited social networks. They mentioned that club activities provided them with opportunities to make friendships with other ethnic groups and relieve stress related to the feelings of isolation. For example, they used similar expressions, such as “I made lots of friends in the book club”; “We were so close and became friends”; and “I never thought that I would make African American friends.” Without engaging in club activities, they believed that there would be a lack of opportunities to have positive interactions with other ethnic groups. Based on the participants’ descriptions, friendships were formed and developed as a result of participation in activities. These relationships helped them to reduce negative feelings such as isolation and loneliness. In addition, they thought that having a shared purpose in the group activities or tasks helped them to enhance feelings of connectedness with others and expand social networks.

Some participants had black belts in Taekwondo that they earned before immigrating. They mentioned that they had performed Taekwondo skills and techniques for the community many times. Presenting these skills and techniques to the community allowed them to experience a level of connectedness with the community through audience-interaction that they previously had not felt. Maintaining Taekwondo practices was helpful for them to relieve their stress.

A few participants volunteered to help others (e.g. older adults and young children) as a way of coping with challenges of living apart from their families. One participant volunteered to help older adults at the senior center because he believed that helping older adults reminded him of his grandparents who lived in Korea. Interacting with older adults at the senior center provided opportunities for him to develop feelings for them as a family. He believed that his feelings arising from the separation from his family were reduced because he was able to treat them like his grandparents.

Another participant had a similar experience. She gave free violin lessons to local children. Interacting with the children enabled her to become part of the community. “With my musical talents, I am so proud to give something back to society. I do not feel like a stranger anymore.” She felt that her sense of belonging came from sharing time, experiences and techniques with children and their parents. In addition, she believed that providing free violin lessons played an important role in relieving her stress and facilitating positive emotion to her, an important coping strategy that she was able to develop.

Another example of a coping strategy included engaging in diverse hobbies. Some participants maintained and developed hobbies such as traveling, drawing, practicing music instruments, and exercise and interests. They believed that doing their hobbies provided a strategy for dealing with daily life stress. For example, one participant said,

Sometimes, I really missed my mom and wanted to go back to Korea … I just played the violin and it gave me excitement in my life.

She believed that playing the violin was a way of avoiding stressful situations and enhancing positive feelings. Some of the participants visited parks and recreation facilities as a way of reducing stress. They mentioned that running and/or walking in the park were stress-relievers because they “recharged” their lives. They felt that the park provided a positive atmosphere where participants coped with stress associated with the exchange adaptation with acculturation process.

Based on the participants’ experiences, they engaged in a variety of meaningful activities as a way of dealing with challenges and stress related to acculturation. They often gained different outcomes associated with a given activity. In addition, engagement in club activities provided participants with opportunities to interact with other ethnic groups and to enhance a sense of enjoyment and happiness in their lives. Overall, the activities provided participants with a sense of belonging and connectedness with others in their community.

### Social support

Social support from friends and family members was another salient theme that emerged from the data. The analysis of the in-depth interview data suggested that participants received social support from their friends and family members, which contributed to development of a coping strategy. In particular, maintaining and developing social relationships with friends provided participants with positive feelings and connectedness with them. In terms of social support from friends, most participants had opportunities to share various immigration experiences and strategies for cultural adaptation with their friends. Their main source of social support was from those who had similar experiences associated with immigration such as other Korean immigration students and/or Asian immigrant students. Most participants thought that they had similar immigration experiences associated with adaptation challenges. By providing a social support system for each other, they were more likely to react to various challenging situations in a collective way. For example, one participant said,

Whenever I am stressed out, I usually hang out with Korean friends. Sometimes we shared lots of things together and those conversations with them really helped me out to deal with lots of stress.

Some participants believed that it was challenging for them to share their stressful situations with American friends because of their differences in ways of thinking and behaving. Even though they developed a certain level of friendship with other ethnic groups, they thought that they were limited in developing a deepened level of friendship because of cultural and ethnic differences. One participant mentioned that his American friends suffered from a lack of information about lives of immigrants and expressed limited cultural understandings of Asian cultures.

Most participants regarded the friendships with other Korean students as important coping resources for dealing with stress because they lived with an American hosting family and experienced a limited amount of quality time with their families in South Korea. They treated their friends like their own family and made concerted efforts to take care of each other as family members would. They thought that social support from their friends played an important role in enhancing a sense of psychological health and reducing negative feelings. One participant mentioned that whenever she experienced challenges such as adaptation to a new school environment or misunderstandings of American cultures, her friends were always supportive to her and provided valuable advice to her. Thus, development and maintenance of strong and intimate friendships provided an important coping strategy for dealing with adaptation challenges.

An interesting discovery was that most participants were reluctant to admit their stressful life events to their parents who live in South Korea. They believed that if they shared some difficulties of adaptation and/or schoolwork with parents, their parents would have become very worried. They mentioned that their parents had sacrificed a great deal in their lives to support them financially and to provide them with an opportunity for their education and it would be disrespectful to cause them worry. This cultural value additionally supported the need for their friends to become their families.

Two participants lived with their parents, and social support from their parents provided an important resource for dealing with stressful life events. The participants mentioned that after immigration, they developed more intimate relationships with their parents. They believed that their parents also experienced numerous life challenges associated with adaptation, which helped them understand their children's lives. Thus, some participants believed that sharing love and supporting each other in a family setting served as an important coping strategy for dealing with stressful events during the adaptation process.

The life experiences of the participants show that social support from their friends and family serves as an important vehicle for coping with stressors and developing intimate relationships with friends and family. In particular, for those who live in a hosting family, the emotional and social support from the same ethnic group of friends plays an important coping resource for dealing with acculturative stress.

### Positive emotion

Positive emotion is a theme that is characterized as a coping resource in regard to the stress-coping among participants. During acculturation, participants experienced positive and negative emotions. In general, they expressed negative emotions as a result of the adaptation process such as difficulties of adaptation to a new school environment, acquiring new culture and language, limited social interactions with other ethnic groups, and schoolwork.

In spite of negative emotions associated with the adaptation process, some participants developed positive emotions and feelings as a coping strategy for dealing with challenges associated with adaptation. They developed the ability to focus on positive traits because they believed that their positive attitudes and emotions helped them to reduce life challenges and barriers and consequently to deal well with stressful situations. Their positive emotions facilitated their abilities to cope with a variety of stressors. Most of them exhibited several expressions that described the development of positive emotions as a process of adaptation such as

I stayed focused on positive minds even though living in American is not easy at all,

Rather than considering stress and negative feelings, keeping positive really helps me a lot,

and

I realized that I had the ability to deal with all challenging situations with the positive mind sets.

Such statements indicate that participants attempted to create positive emotions with positive thoughts.

Some participants believed that challenges associated with acculturation provided new opportunities to facilitate their personal growth and enhance a sense of positive feeling, which helped reduce levels of life stress. Some of them thought that they became much stronger mentally through the adaptation process. Such positive emotions and attitudes served as an important coping strategy for participants because they believed that through stressful life events they developed their positive emotions as a buffer against them.

Participants indicated that positive emotion increased with the realization of personal strength and talent, which served as another coping skill. Using personal strengths and talents, some participants experienced positive emotions because they realized that they had the ability to overcome challenges and enjoy their lives. Some participants realized that becoming aware of their strengths and talents generated excitement, enjoyment, and self-esteem, and constituted a strategy for coping with stress of acculturation. Those positive outcomes facilitated participants’ positive emotions. For example, one participant stated that performing on stage for theater made her happy because she “overcame the language issue and am pursuing my dream of being an actress.” She mentioned that pursing her career based on her talent of acting developed positive emotion as a source of her coping skill. Another participant believed that he had earned respect from the individuals in his class while teaching Taekwondo. Through volunteering to teach Taekwondo he experienced feelings of excitement and happiness that helped to reduce negative life stress.

Based on the participants’ experiences and statements, the adaptation process is associated with not only negative feelings but also positive feelings and emotions associated with enjoyment, happiness, and excitement. Facing the challenging circumstances related to the adaptation process, participants were able to develop the ability to deal with challenges and become aware of their strengths and talents as facilitators of a sense of positive emotion and coping resources for dealing with stressful situations.

## Discussion

Throughout this article, we explored how Korean immigrant adolescents perceived and coped with the stress associated with immigration experiences. The thematic analysis provided rich examples of coping strategies and coping resources that Korean immigrant adolescents utilized and developed. We gained information and knowledge about the cultural impact on Korean immigrant adolescents’ stress and health. The cultural background and life circumstances (e.g. living apart from parents and adaptation difficulties) of Korean immigrant adolescents are closely associated with acculturative stress. Participants developed their own specific strategies for dealing with stressful life events and facilitating positive emotions.

Previous research has demonstrated that activities in which adolescents participate contribute to interpersonal development via connections with others that promote interpersonal relationships, which, in turn, help expand the social networks (Eccles & Templeton, 2002; Holland & Andre, 1987; Patrick et al., 1999). In addition, involvement in activities provides a unique context through which adolescents develop social relationships and better understanding of their peers from culturally and ethnically diverse groups (National Research Council, 2000; Patrick et al., 1999). This article supports the body of knowledge that Korean immigrant adolescents develop interpersonal relationships and expand their social networks through activity involvement. The finding of this article indicates that activity participation plays an important role in dealing with negative feelings (e.g. social isolation and loneliness) because of positive social interactions with others.

This article supports the previous studies that Korean immigrant adolescents engaging in a volunteering activity developed feelings of social connections with the community. According to Yates and Youniss (1996), adolescents participating in a volunteering activity developed interpersonal skills and social relationships that encouraged a sense of belongingness and connectedness to the community. Other related studies have shown that having a sense of belongingness and responsibility within a community service activity reduces feelings of social alienation as well as increases empathy, openness and tolerance toward others and promotes positive social interactions (McKinney, 2002; Yates & Youniss, 1996). The finding of this study demonstrates that such a sense of belonging combined with relationships with others in the community serves as an important coping strategy.

The findings of this article suggest that culture plays an important role in creating coping strategies such as collectivism for Korean immigrant adolescents. Participants exhibited collectivistic behaviors as an expression of social support and shared effective adaptation skills with each other. They developed more close and intimate friendships, which contributed to their adaptation and coping skills. Additionally, the event of immigration provided Korean immigrant parents with opportunities to develop better relationships with their children. Social support from parents played an important role in reducing negative feelings and stress for participants.

The findings of this study show that leisure activities provided participants with opportunities to overcome the challenges and obstacles associated with activity participation as well as adaptation processes and use these experiences to create a strong mind and a sense of happiness. A few studies have examined the value of activity participation as a way of coping with stress and concluded that participation in activities plays an essential role in coping with stressful experiences and leads to resilience and growth (Chun & Lee, 2008; Griffin, 2005; Hutchinson, Loy, Kleiber, & Dattilo, 2003; Iwasaki & Barlett, 2006). Thus, the results of this study extend the value of activity participation as a way to cope with acculturative stress and lead to positive emotions among Korean immigrant adolescents.

This study is aligned with the concept of Positive Psychology and stress-related growth among immigrant adolescents. A growing body of knowledge provides evidence that individuals experience positive psychological changes following stressful life events (Calhoun & Tedeschi, 1999; Tedeschi & Calhoun, 1995). The conceptualization is supported by Positive Psychologists who have demonstrated that people have the capability and strength to prevent or lessen the damage of disease, stress, and disorder despite negative aspects of life (Folkman & Lazarus, 1980; Gable & Haidt, 2005). While in the middle of the stressful adaptation process, Korean immigrant adolescents developed a sense of psychological thriving through positive emotion and positive feelings. These individuals utilized positive emotions and positive feelings as coping strategies that contributed to psychological thriving. In this study, Korean immigrant adolescents utilized their own strengths and talents as their coping strategies to enhance a sense of happiness and positive emotions. Positive emotions and qualities serve as an important resource for coping with acculturative stress among Korean immigrant adolescents.

Overall, this article has provided what we consider to be important findings for health professionals. We explored the stress strategies of Korean immigrant adolescents and based on the stress-coping strategies, health professionals can have a better understanding of the stress-coping strategies among Asian immigrant adolescents. Some immigrant adolescents may face adaptation difficulties and challenges, which negatively affect the perception of health and well-being. In addition, this article is helpful for health professionals to developing a sense of multicultural competence with cultural knowledge and to create effective coping resources of considering the value of activity participation, social support from peers, and psychological functions for immigrant adolescents. Thus, health professionals are better able to provide Asian immigrant adolescents who are struggling with acculturation effective programs that contribute to a reduction of negative feelings and acculturative stress.

### Limitations and need for future research

This study has some limitations that need to be noted. First, we examined the stress-coping among Korean immigrant adolescents only. Other ethnic immigrant adolescents may develop their own coping strategies and resources and their own cultures may be involved in stress-coping. Future research is needed to examine how other immigrant adolescents perceive and cope with acculturative stress.

Participation in meaningful activities plays an important role in reducing acculturative stress among Korean immigrant adolescents. Many researchers (Chun & Lee, 2008; Griffin, 2005; Hutchinson et al., 2003; Iwasaki & Barlett, 2006) have found that participation in leisure serves as an important resource for dealing with stressful life events. However, there is little information on how immigrant adolescents utilize their leisure time for dealing with acculturative stress and leading to personal growth and resilience. Future study should include examination of the relationship between leisure activities and associated outcomes among immigrant adolescents.

We examined only the coping strategies and resources of Korean immigrant adolescents utilized for dealing with the stress from acculturation. There may be additional benefits (e.g. psychological health, social health, and physical health) that result from these coping strategies. It may be helpful for future researchers to examine the benefits of coping strategies focused on health and well-being among immigrant adolescents.

Furthermore, we explored the stress-coping of Korean immigrant adolescents after immigration. We did not examine the difference of coping strategies after and before immigration. It may be interesting for future researchers to investigate similarities and differences in coping strategies between Korean immigrant adolescents and Korean adolescents.

### Implications

Theoretically, based on the findings of this article, we offer some suggestions on how to effectively address a variety of stressors among Asian immigrant adolescents. The first suggestion is to create and provide a variety of meaningful activities that encourage Asian immigrant adolescents’ participation. In general, Asian immigrants experience limited participation in activities. For example, when comparing two groups of Asian immigrants and Asian Americans born in the United States, Asian immigrants reported that they participated in leisure time physical activities at a much lower rate than Asian Americans (Kandula & Lauderdale, 2005). Based on these findings, specific cultural characteristics such as adaptation process and cultural conflicts may be associated with healthy leisure behaviors of immigrants. Thus, it is important for community service providers to create and develop strategic plans that encourage Asian immigrant adolescents to engage in such as volunteerism and club activities.

Second, Asian immigrant adolescents experience specific cultural values and beliefs associated with collectivism and family-oriented values. Such culture-specific values have an impact on these individuals’ decisions and lives because social support from the same ethnic groups has a significant impact on the coping resources for Asian immigrant adolescents. Health care service providers need to create counseling programs that are sensitive to culture-related matters so that they provide effective interventions for Asian immigrant adolescents who experience adaptation difficulties.
